# Delphi consensus validation of an integrated climate sensitive diabetes management model for climate vulnerable health systems in Iran

**DOI:** 10.1038/s41598-026-56784-z

**Published:** 2026-06-08

**Authors:** Amir Ghasemian, Elaheh Hooshmand, Ehsan Mousa Farkhani, Mansoureh Kouhi, Mehrdad Salehi, Seyed Saeed Tabatabaee, Javad Moghri

**Affiliations:** 1 Student Research Committee, Mashhad University of Medical Sciences, Mashhad, Iran; 2 Social Determinates of Health Research Center, Mashhad University of Medical Sciences, Mashhad, Iran; 3 Department of Epidemiology, School of Health, Mashhad University of Medical Sciences, Mashhad, Iran; 4 Research Institute of Meteorology and Atmospheric Science, Climatological Research Institute (CRI), Mashhad, Iran; 5 Department of Management Sciences and Health Economics, School of Health, Mashhad University of Medical Sciences, Mashhad, Iran

**Keywords:** Type 2 diabetes, Climate change, climate-resilient health systems, Delphi technique, Health-system modelling, Low- and middle-income countries, Iran, Climate sciences, Health care

## Abstract

Type 2 diabetes mellitus (T2DM) is rising rapidly in Iran, where climate-related hazards such as heatwaves, sand and dust storms, and disruptions to food and medicine supply chains challenge continuity and quality of care. We sought to validate a climate-sensitive, health system-oriented management model for T2DM using a Delphi consensus process. Eighteen experts in diabetes, noncommunicable diseases and health systems rated 51 statements derived from the preliminary Integrated Iranian Climate-Sensitive Diabetes Management Model (ICDMM), mapped onto six domains aligned with WHO health system building blocks. Items were scored on a binary agree/disagree scale, with a ≥ 70% agreement threshold for consensus. In round 1, agreement ranged from 61.1% to 100.0%; 46 statements met the threshold and five were revised. In round 2 all revised items achieved ≥ 80% agreement, and ultimately all 51 statements reached consensus. The validated ICDMM comprises six domains and 51 components spanning climate-informed governance, resilient and equitable service delivery, workforce preparedness, digital and early-warning information systems, climate-resilient supply chains, and equity-oriented climate-sensitive financing. The ICDMM offers a disease-specific framework for operationalising climate-resilient health system principles for T2DM in a climate-vulnerable middle-income setting and now requires implementation and evaluation in real-world practice.

**Supplementary Information:**

The online version contains supplementary material available at 10.1038/s41598-026-56784-z.

## Introduction

Type 2 diabetes mellitus (T2DM) is an escalating global health challenge, with prevalence projected to surpass 1.3 billion by 2050 due to aging populations, sedentary lifestyles, and dietary transitions^1^. Simultaneously, climate change—recognized by the World Health Organization as the greatest health threat of the 21st century—disrupts healthcare delivery through intensified heatwaves, floods, and storms^2,3^. Mounting evidence links climatic stressors to higher T2DM incidence, complications, and healthcare demand^4,5^.

Rising temperatures, extreme weather, air pollution, and food insecurity collectively alter diabetes risk and management pathways. Heat exposure induces dehydration and insulin resistance, impairing glycemic control^6^, while air pollution—identified as a major contributor to the global burden of T2DM—further heightens cardiometabolic risk^7^. In addition, climate-driven agricultural disruptions (e.g. droughts, floods) and rising food prices push vulnerable populations toward low-cost, ultra-processed diets, worsening nutritional quality and glycemic outcomes^8^. These converging factors are reshaping the epidemiology of diabetes and challenging conventional care models.

In Iran, these global trends find acute expression. National data indicate that adult diabetes prevalence stood at approximately 14.15% in 2021—a roughly 45% increase from five years earlier—equating to nearly 5.7 million Iranians with diabetes in 2022 and a projected burden exceeding 9 million by 2030^9^. At the same time, the country faces intensifying climate stressors: increasing drought frequency, more severe heatwaves, water scarcity, and livelihood-threatening pressures on food production^10^. Southern provinces such as Khuzestan, Hormozgan and Sistan and Baluchistan—regions characterized by extremely high summer temperatures, recurrent sand/dust storms, food insecurity, and relatively fragile health infrastructure—are particularly vulnerable to the twin burden of chronic disease and climate stressors^11^. These dynamics complicate the provision of quality diabetes care and amplify the vulnerability of at-risk populations.

Heatwaves exemplify how climate extremes can acutely worsen diabetes outcomes. Extreme heat exposure induces dehydration and elevates insulin resistance in people with diabetes^6^, often triggering hyperglycemic crises and other complications. Empirical studies have documented spikes in diabetes-related hospital admissions during heat events—for instance, in Brazil a 5 °C increase in daily mean temperature was associated with a 6% rise in hospital admissions for diabetes in subsequent days^12^. Moreover, climate disasters such as floods and dust storms disrupt access to health programmes, medication supply chains, power grid stability (with knock-on effects for insulin refrigeration), and health-record systems. These disruptions have been linked with deteriorations in glycemic control and increased incidence of diabetic emergencies^13,14^.

Despite advances in global diabetes-management frameworks, relatively few models have explicitly incorporated climate-change adaptation within their architectures, and even fewer are tailored to the needs of middle-income settings such as Iran^15–17^. While WHO and International Diabetes Federation (IDF) toolkits provide robust evidence-based strategies for T2DM control, their practical implementation in climatically stressed, resource-constrained health systems often falls short. Structural weaknesses in Iran—including fragmented governance, under-resourced primary care, and underdeveloped information systems—further undermine the resilience of diabetes care under climate stress^18–20^. These realities highlight an urgent need for an evidence-based, locally contextualized, climate-informed diabetes-management model to bolster Iran’s health-system preparedness for environmental change.

However, most existing guidance remains either disease-agnostic or narrowly focused on clinical management, with limited attention to how climate hazards interact with health-system structures, financing mechanisms, and service delivery for diabetes. Recent climate–health frameworks and scoping reviews describe broad pathways and policy options, but they rarely translate these into an integrated, operational model that specifies what ministries of health, providers, and financing agencies should do differently for T2DM under climate stress in specific national contexts^5,15–17^. To our knowledge, no published framework has systematically adapted global climate-resilient health-system principles into a diabetes-specific operational model for the realities of Iran’s health system and its climate-vulnerable southern provinces.

To substantiate this claim of novelty, we explicitly contrast the ICDMM with the most relevant existing frameworks. The WHO Operational Framework for Building Climate Resilient and Low Carbon Health Systems^21^ provides high-level guidance across six building blocks but remains disease‑agnostic and does not prescribe concrete actions for diabetes care. The International Diabetes Federation (IDF) has published general recommendations on climate change and diabetes, yet these lack health‑system operational steps, financing mechanisms, or Delphi‑based validation. Similarly, recent scoping reviews^5,22^ identify broad adaptation strategies but do not translate them into a tested, component‑level model for a specific chronic disease. In contrast, the ICDMM translates climate‑resilient principles into 51 action‑oriented statements across the same six WHO building blocks, with specific innovations – climate‑sensitive financing, psychosocial resilience, AI‑enabled surveillance, and equity‑focused governance – that are absent or only superficially treated in existing guidance. Furthermore, the ICDMM is part of an emerging set of climate‑sensitive models that have undergone formal Delphi content validation in a middle‑income country context, providing a level of expert consensus that generic frameworks typically lack.

To address this gap, we designed a three-phase research programme to develop an Integrated Iranian Climate-Sensitive Diabetes Management Model (ICDMM) tailored to the national health system and climate context. Phase 1 comprised a scoping review of international and regional diabetes-management models to map existing approaches and identify gaps in climate adaptation. Phase 2 used qualitative interviews with key stakeholders—including endocrinologists, general practitioners, service managers, disaster-preparedness specialists, and health-policy officials from climate-vulnerable provinces—to elucidate contextual barriers, system needs, and opportunities for climate-smart diabetes care. Drawing on the combined evidence from these phases, we constructed a preliminary ICDMM organised around six domains aligned with the WHO health-system building blocks:^1^ leadership and governance^2^, service delivery^3^, health workforce^4^, health information systems^5^, medical products, vaccines and technologies, and^6^ health financing^23^.

This paper reports the formal content validation of the ICDMM through a structured two-round Delphi process with multidisciplinary national experts. By focusing on Iran’s climate-vulnerable health system and specifying climate-sensitive actions across all WHO health-system building blocks, the ICDMM offers a context-sensitive framework for managing T2DM under the pressures of climate change, translating global climate-resilient health-system principles into disease-specific operational actions. To our knowledge, this is one of the few Delphi-validated, climate-sensitive, health system–oriented models developed specifically for type 2 diabetes in a middle-income country.

The validated model is expected to strengthen Iran’s readiness to tackle both chronic-disease burdens and climate-related risks, and to contribute to the global agenda of building climate-resilient health systems capable of delivering sustainable, equitable chronic-disease care in a warming world. Specifically, this Delphi study aimed to^1^ assess the face and content validity of each proposed component of the Integrated Iranian Climate-Sensitive Diabetes Management Model (ICDMM), and^2^ refine statements that did not initially reach consensus based on structured expert feedback across two Delphi rounds.

## Methods

### Study design

This Delphi study formed the culminating phase of a broader research programme focused on developing and validating a climate-sensitive management model for type 2 diabetes in Iran. In this phase, we implemented a two-round, cross-sectional Delphi consensus exercise to refine and validate the proposed model in the context of climate change. The Delphi technique was chosen as an established approach for structuring expert judgement on complex, multidimensional questions and for enabling repeated reflection and feedback across rounds until convergence of views is reached^24,25^.

The study was designed and reported in accordance with established methodological guidance, including the ACCORD guideline for consensus methods, to maximise transparency and reproducibility^26^. A completed ACCORD reporting checklist is provided in Additional File 1. Key ACCORD elements—including a priori specification of the consensus threshold and maximum number of rounds, anonymised responses, controlled feedback between rounds, and transparent reporting of panel selection and attrition—were incorporated into the design. The theoretical foundation drew on the World Health Organization (WHO) health system building blocks and related frameworks for climate-resilient health systems, which emphasise the interdependence of service delivery, governance, health workforce, information systems, medical products, vaccines and technologies, and health financing^17,20,23^. The Delphi protocol was not prospectively registered on a public registry. The study was led by a steering group comprising experts in health systems, epidemiology, climate and environmental health, and diabetes care (SST, JM, EH, EMF, MK), who oversaw model development and the Delphi process.

## Development of the Delphi questionnaire

The preliminary climate-sensitive type 2 diabetes management model was constructed in earlier phases of the research programme, drawing on two main evidence sources: (i) a scoping review of international and regional diabetes-management and climate–health frameworks, and (ii) a qualitative study with key stakeholders in climate-vulnerable provinces of Iran. In the first step, the main categories and subcategories identified in the scoping review were mapped onto the six WHO health system building blocks. In the second step, themes and subthemes from the qualitative analysis (e.g. continuity of care during climate events, workforce deployment, supply-chain vulnerabilities, financial protection and equity concerns) were systematically compared with this map in a matrix to identify convergent, complementary and missing components.

Overlapping concepts were merged, and each distinct component was rewritten as a clear, action-oriented statement. Through an iterative team-based review, the long-list of candidate components was refined for clarity, non-redundancy and feasibility, resulting in 51 statements organised across the six WHO-aligned domains:^1^ leadership and governance^2^, service delivery^3^, health workforce^4^, health information systems^5^, medical products, vaccines and technologies, and^6^ health financing. Each statement represented a proposed requirement of a climate-sensitive type 2 diabetes management model within the Iranian health-system context. A summary matrix showing the linkage between scoping-review domains, qualitative themes and Delphi statements is provided in Supplementary Table S1. A brief summary of the underlying evidence and the matrix of components (Supplementary Table S1) were provided to panel members alongside the Delphi questionnaire to support informed judgement.

Each item was rated on a binary response scale (“agree” / “disagree”) to enable a clear and quantifiable assessment of consensus. We selected this simple dichotomous format to minimise respondent burden and to provide a clear distinction between endorsed and non-endorsed components, which was appropriate given that the primary aim of this Delphi was to confirm or reject candidate model elements rather than to rank their relative importance; this strategy is commonly used in Delphi studies that prioritise feasibility and clarity over fine-grained ranking^24,27–29^. We acknowledge that this binary approach, while facilitating clarity and reducing respondent burden, may have obscured finer distinctions between “essential” and “desirable” components and may have marginally inflated the perception of consensus. Nevertheless, the dichotomous scale was deemed appropriate for a content‑validation Delphi where the goal was to confirm inclusion or exclusion of conceptual items rather than to rate their relative importance. Future iterations of the ICDMM could adopt a Likert-type scale to explore granular levels of importance or urgency for each model component. A free-text field at the end of the questionnaire allowed panel members to provide comments, justifications, or suggestions for rewording, adding, or merging statements. Prior to distribution, the face and content validity of the questionnaire were reviewed by three independent experts in diabetes care and health systems research, and minor modifications were made to improve clarity, relevance, and conceptual coherence^30,31^.

## Participants

Purposive sampling was used to recruit a multidisciplinary panel of experts spanning diabetes care, non-communicable disease (NCD) control, health-systems management, and climate-related health planning. In total, 30 experts were invited. The target sample of approximately 30 invitees was chosen to balance multidisciplinary breadth with the feasibility of repeated participation, and panel members were selected by the steering group based on these criteria. Eighteen experts (60.0%) completed the first-round questionnaire and were included in the Delphi panel; all 18 also completed the second round, yielding a 100% retention rate between rounds.

Although the final expert panel size (*n* = 18) was modest relative to some larger Delphi studies, it falls within the widely accepted range for focused content validation exercises, which typically include 10–30 participants, particularly when the expertise is highly homogeneous and the study is conducted within a single-country context^24,29^. In this study, the panel composition was intentionally designed to provide strong technical depth across diabetes care, climatology, health systems, and disaster management, drawing experts from national and provincial health authorities, universities, and climate–health working groups. This multidisciplinary yet specialised structure was considered appropriate for evaluating complex cross-sectoral issues where informed judgment is more critical than broad representativeness. The high retention rate across all rounds (100%) and the absence of missing data further enhanced the internal validity and stability of consensus. Nonetheless, the panel size remains a methodological limitation, and the absence of patient representatives and frontline community health workers highlights an important gap that could be addressed in future validation phases through participatory or community-engaged approaches.

The final panel comprised policymakers, health-services managers, endocrinologists and general practitioners, epidemiologists, and specialists in environmental health, health in disasters and emergencies, and health policy from different universities of medical sciences and health authorities across Iran, encompassing both national- and provincial-level perspectives.

Experts were initially identified through national and provincial NCD and diabetes committees, MOHME working groups on climate and health, and academic networks in climatology, health-systems research and disaster risk management. Where appropriate, a snowball strategy was also used, whereby initial invitees suggested additional experts who met the eligibility criteria. Participant characteristics (age, gender, educational level, field of expertise, and years of professional and diabetes-related experience) are summarised in Table 1. Patients and members of the public were not involved in this Delphi exercise, which focused on expert stakeholders within the health system.

Eligibility criteria were: (a) at least 5 years of professional experience in diabetes care, NCD programmes, climate-related health planning, or health system management; (b) possession of at least a bachelor’s degree in a relevant discipline; and (c) willingness to participate in both Delphi rounds.

## Data collection and analysis

A two-round Delphi process was conducted using self-administered questionnaires sent electronically, enabling participation from geographically dispersed experts while maintaining confidentiality. Each participant was assigned a unique study ID, questionnaires were returned without personal identifiers, and only aggregated round-level results were fed back to the panel. Feedback between rounds was provided in aggregate form (group-level agreement percentages for each statement), so that individual responses were not visible to other panellists. No financial or other incentives were offered for participation. No specific accessibility adaptations were required because all panel members were health professionals with routine access to email and online surveys. The two Delphi rounds were conducted between September 2025 and November 2025.Following common practice in modified Delphi studies and to minimize participant burden, we pre-specified a maximum of two rounds, with the option of a third round or item exclusion if consensus was not reached^24,26^.

In the first round, the questionnaire was emailed to all 30 invited experts. The first-round questionnaire remained open for approximately two weeks, during which participants received two reminder e-mails (after 7 and 12 days) to encourage completion. The second-round questionnaire was distributed three weeks after closure of Round 1 and remained open for a similar two-week period with one reminder e-mail midway through the response window. Participants were asked to indicate their agreement with each statement and to provide optional written comments or suggestions for refinement. Responses were summarised using descriptive statistics (frequencies and percentages of “agree”), and an a priori consensus threshold of ≥ 70% agreement was defined for this round. This cut-off was chosen in line with widely used thresholds in health-related Delphi studies, where levels between 70% and 80% agreement are typically considered indicative of consensus^24,27–29^.

Based on the first-round results and expert feedback, five statements with the lowest levels of agreement or the greatest conceptual ambiguity were revised to improve precision, remove redundancy, and enhance alignment with the underlying model. Statements that had already reached the consensus threshold were retained unchanged. The revised questionnaire, which included these five modified items together with a summary of first-round agreement levels for all statements, was then redistributed via email to the same panel in the second round. Experts were invited to reconsider their initial responses in light of the group results and to record their final opinions. The aim of the second round was to confirm consensus on the revised items and to stabilise the panel’s overall judgements. No third round was required because all items achieved the predefined consensus threshold by the end of Round 2, and no missing data occurred as only complete questionnaires were included in the analysis. No major deviations from the pre-specified Delphi protocol occurred.

Qualitative feedback from both rounds was analysed in parallel with the quantitative results. All free-text comments were exported to an Excel matrix and independently reviewed by two members of the research team, who thematically grouped suggestions such as clarifying target populations, specifying climate-related risk conditions, and identifying responsible actors. Where needed, they proposed alternative wording or consolidation of overlapping statements, and disagreements were resolved through discussion with a third senior researcher. This process informed the rewording of items that had low agreement or were judged conceptually ambiguous after Round 1.

Data were analysed using SPSS version 26. Consensus levels were categorised following established Delphi standards as strong (> 85% agreement), moderate (70–84%), and low (< 70%), with low-consensus items revised and re-submitted in the subsequent round^32–34^. For each statement, the percentage of endorsement (“agree”) was calculated, and statements with ≥ 70% agreement in the final round were considered to have achieved consensus. Given the dichotomous response format, analyses were confined to descriptive statistics. Inferential measures such as Kendall’s W or the Friedman test, which are designed for ranked or Likert-scale data, were not applied. Instead, agreement was assessed using frequencies and percentages, consistent with methodological recommendations for Delphi studies employing binary response options^33,34^.

## Operationalisation of ICDMM components for practical implementation

While the Delphi consensus establishes content validity, the ultimate value of the ICDMM lies in its ability to guide practical design and implementation. To explicitly address this, we have mapped the 51 validated statements to concrete operational steps. Supplementary Table S3 provides an operationalisation framework for ten key components – including the five items that required revision after Round 1 (psychosocial resilience, workforce distribution, supply chain stability, insurance coverage, and equity monitoring) and five items with the highest consensus (100% agreement). For each component, the table specifies: (i) suggested responsible actor(s) at national, provincial, or facility level; (ii) initial implementation steps with realistic timelines (e.g., 3 months, 6 months, 1 year); and (iii) measurable process indicators to monitor progress. This operational framework is designed to help health system managers, policymakers, and researchers translate consensus statements into actionable programmes, thereby addressing a key limitation of purely consensus-based models. The full table is provided in the supplementary material.

### Ethical considerations

The study protocol was reviewed and approved by the Ethics Committee of Mashhad University of Medical Sciences (approval code: IR.MUMS.FHMPM.REC.1403.098). All ethical principles were observed throughout the study. Participation was entirely voluntary, and returning a completed questionnaire was taken as implied informed consent. All data were anonymised prior to analysis, and findings are reported in aggregate form only, without personal identifiers. The study followed the principles of privacy, confidentiality, and the Declaration of Helsinki^35^.

## Results

### Participant characteristics

Eighteen experts completed both Delphi rounds and were included in the analysis. Socio-demographic and professional characteristics of the panel are summarised in Table 1.

**Table 1:** Demographic and professional characteristics of Delphi experts (*N* = 18).

Characteristic	Category / value	*n*	%
*Socio-demographic characteristics*			
Age (years)	Mean ± SD (range): 45.1 ± 7.5 (34–58)	18	–
Gender	Male	12	66.7
	Female	6	33.3
*Professional characteristics*			
Years of professional experience	Mean ± SD (range): 17.3 ± 7.4 (7–30)	18	–
Highest educational level	Doctorate	15	83.3
	MSc	2	11.1
	BSc	1	5.6
Field of expertise	Health services management	6	33.3
	General practitioner	4	22.2
	Health in disasters	3	16.7
	Health policy	2	11.1
	Environmental health	1	5.6
	Public health	1	5.6
	Endocrinology subspecialist	1	5.6

### Delphi consensus process

The Delphi questionnaire included 51 statements reflecting components of the Integrated Iranian Climate-Sensitive Diabetes Management Model (ICDMM), organised across six domains aligned with the WHO health system building blocks (leadership and governance, service delivery, health workforce, health information systems, medical products, vaccines and technologies, and health financing). All items were rated on a binary “agree/disagree” scale.

In the first round, levels of agreement ranged from 61.1% to 100.0%. Overall, 46 of the 51 statements (90.2%) met or exceeded the predefined consensus threshold of ≥ 70% agreement and were therefore retained. Five statements that did not reach the threshold received qualitative feedback from the experts and were subsequently revised. In the second round, the revised statements were resubmitted to the panel together with a summary of the first‑round results for all items. Following this iteration, the remaining five statements achieved ≥ 70% agreement. Consequently, all 51 statements reached the predefined consensus level across the two Delphi rounds and were retained in the final model.

The five statements revised after the first round addressed the following issues:

- Strengthening psychosocial resilience of people with type 2 diabetes and their families in the face of climate-related crises (66.7% agreement in Round 1);
- Fair distribution of, and support for, specialised diabetes workforce in climate high-risk areas (61.1%);
- Stable and reliable supply of diabetes medicines and devices during climate-related crises (66.7%);
- Adequate insurance coverage and financial protection for people with type 2 diabetes in climate high-risk situations (66.7%);
- Monitoring equity in payments for type 2 diabetes care and improving access and continuity of services (61.1%).

Based on written comments, these five statements were reworded to clarify their scope (e.g. explicit reference to climate-related risk conditions), strengthen their operational focus, and reduce ambiguity regarding target populations and responsible actors. All other items, which had already achieved consensus, were kept unchanged. Comments were exported to an Excel matrix and independently reviewed by two members of the research team, who thematically grouped suggestions (for example, clarifying target populations, specifying climate-related risk conditions, and identifying responsible actors) and reached consensus on the required modifications; any disagreements were resolved through discussion with a third senior researcher. Most comments requested more explicit reference to climate-related risk conditions, clearer distinction between patients, families and staff, and more precise specification of policy or programme levers, and the revised wording addressed these concerns.

Across the free‑text comments provided by the 18 panellists, three main themes recurred consistently, irrespective of whether the comment pertained to a revised or an already‑accepted item. First, experts requested clearer specification of climate‑related risk conditions (e.g., differentiating between acute events such as heatwaves and dust storms versus chronic stressors such as drought or food insecurity). Second, panellists emphasised the need to distinguish between support for different target groups – patients, family caregivers, and health workers – as each faces distinct vulnerabilities during climate‑related disruptions. Third, many comments called for explicit identification of responsible actors (e.g., Ministry of Health, provincial health authorities, insurance organisations, or non‑governmental organisations) to enhance accountability and operational feasibility. These themes informed not only the revision of the five low‑consensus items but also minor wording adjustments in several accepted statements to improve clarity, although no further items required re‑administration.

In the second round, the revised questionnaire was redistributed electronically to the same 18 experts, accompanied by a summary of Round 1 agreement levels. Agreement in Round 2 ranged from 83.3% to 100.0%. All five revised statements surpassed the ≥ 70% consensus threshold, with final agreement levels of 83.3–94.4% (psychosocial resilience: 83.3%; specialised workforce distribution in high-risk areas: 88.9%; stable and reliable supply of diabetes medicines and devices during crises: 94.4%; insurance coverage and financial protection in climate high-risk situations: 88.9%; monitoring equity in payments and improving access and continuity of care: 83.3%).

Taken together, all 51 statements (100%) achieved the predefined consensus threshold by the end of Round 2. Most items fell within the “strong consensus” category (> 85% agreement), with only a small subset—particularly those concerning psychosocial support, workforce distribution, and financial protection—transitioning from low or moderate agreement in Round 1 to consensus after targeted revision. To improve readability, a condensed summary of agreement levels across the six ICDMM domains is presented in Table 2, while the detailed Delphi ratings for all 51 statements, including Round 1 and Round 2 agreement levels and final status, are provided in Supplementary Table S2.

**Table 2:** summary of agreement levels across the six ICDMM domains.

WHO domain	Number of items	Round 1 agreement range (%)	Round 2 agreement range (%)	Final consensus (%)
Leadership & governance	8	83.3–100	–	100
Service delivery	9	66.7–100	83.3	100
Health workforce	8	61.1–100	88.9	100
Health information systems	9	94.4–100	–	100
Medical products, vaccines & technologies	8	66.7–100	94.4	100
Health financing	9	61.1–100	83.3–88.9	100

### Patterns of consensus

The domain of Health Information Systems showed the highest overall agreement in Round 1, with all nine items reaching between 94.4% and 100% – notably, one item (use of artificial intelligence and data mining) achieved 94.4%, while the remaining eight items achieved 100%. Leadership and Governance followed closely, with a range of 83.3–100% and no items below the consensus threshold. The lowest initial agreement was observed in Health Financing (two items at 61.1% and 66.7%) and Medical Products, Vaccines & Technologies (one item at 66.7%), followed by Service Delivery (one item at 66.7%) and Health Workforce (one item at 61.1%). Notably, all five items that required revision after Round 1 shared a common theme: they related to cross-cutting, “soft” system functions involving equity, psychosocial support, and supply chain reliability – specifically: fair distribution of specialised workforce (Health Workforce, 61.1%), psychosocial resilience of patients and families (Service Delivery, 66.7%), stable and reliable supply of diabetes medicines and devices (Medical Products, 66.7%), adequate insurance coverage and financial protection (Health Financing, 66.7%), and monitoring equity in payments (Health Financing, 61.1%). The fact that these five items – but not more clinically or technically focused items – initially fell below the consensus threshold suggests that experts found them conceptually important but initially ambiguous regarding operational responsibility and implementation scope. After targeted revision to clarify climate-related risk conditions, target populations, and responsible actors, all five achieved ≥ 83.3% agreement in Round 2 (psychosocial resilience: 83.3%; workforce distribution: 88.9%; medicine supply: 94.4%; insurance coverage: 88.9%; equity monitoring: 83.3%).

### Validated climate-sensitive type 2 diabetes management model

Following completion of the two Delphi rounds and achievement of full (100%) expert consensus, the Integrated Iranian Climate-Sensitive Diabetes Management Model (ICDMM) was confirmed as a coherent, climate-sensitive, health system–oriented framework for type 2 diabetes care in Iran. The final model comprises six interdependent domains and 51 validated components, explicitly aligned with the WHO health system building blocks.

**Fig. 1:**
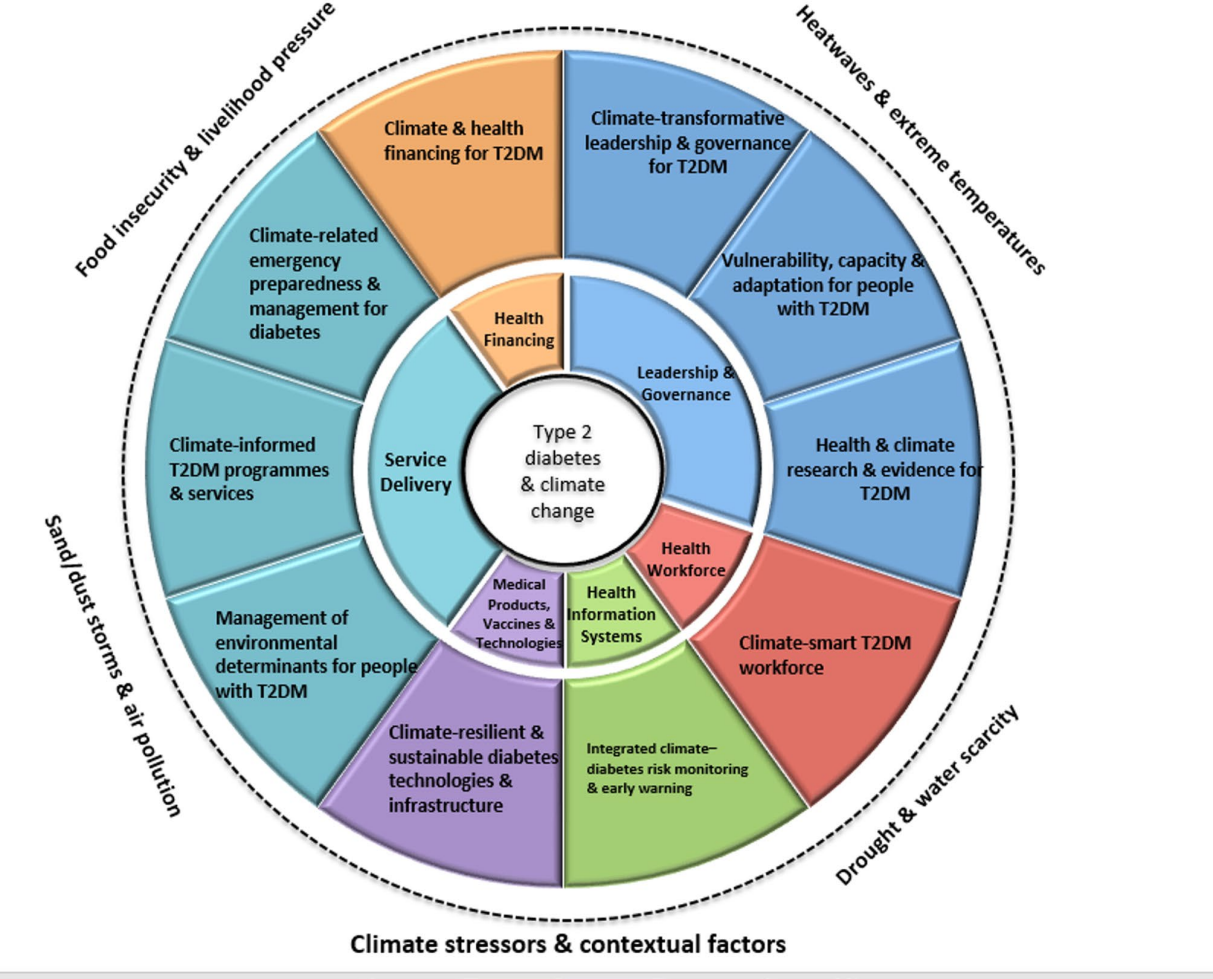
Integrated Iranian clima te-sensitive diabetes management model (ICDMM).

Together, these domains connect climate-informed and equity-oriented leadership and governance, resilient and people-centred service delivery, a trained and supported health workforce, robust digital and climate-aware information systems, secure and sustainable supply chains for medicines, vaccines and technologies, and flexible, innovative health financing arrangements that protect vulnerable patients during climate-related shocks. Experts emphasised that the strength of the ICDMM lies in the vertical and horizontal integration of these components across the health system, enabling coordinated preparedness, response and recovery in the face of climate stressors. The final validated model is summarised in Table 2 and illustrated schematically in Fig. 1.

Figure 1. Integrated Iranian Climate-Sensitive Diabetes Management Model (ICDMM). The model places type 2 diabetes and climate change at the center. Surrounding this core, six domains aligned with the WHO health system building blocks (leadership and governance, service delivery, health workforce, health information systems, medical products, vaccines and technologies, and health financing) represent the health system capacities required to deliver resilient and equitable diabetes care under climate stress. An outer layer of climate and contextual stressors (e.g. heatwaves, drought and water scarcity, sand/dust storms and air pollution, and food insecurity) illustrates how environmental pressures interact with all components of the health system.

## Discussion

The two-round Delphi exercise resulted in high and ultimately unanimous endorsement of all proposed ICDMM components, with every one of the 51 statements reaching the ≥ 70% consensus threshold by Round 2 and most items achieving > 83% agreement. This high level of consensus indicates that the multidisciplinary expert panel considered the model conceptually coherent, contextually relevant, and aligned with existing climate–health and diabetes care priorities in Iran. At the same time, consensus should be interpreted as agreement on perceived relevance rather than proof of feasibility or effectiveness in real-world settings.

Only a limited number of items—primarily those related to psychosocial resilience, workforce distribution, supply chain stability, and financial protection—required modification between rounds. The need for refinement in these domains suggests that while the overall structure of the ICDMM was viewed as appropriate, greater operational clarity was required for components involving cross-sectoral coordination, financing mechanisms, and non-clinical support systems.

Importantly, the convergence of expert opinion across all six WHO-aligned domains strengthens the model’s face and content validity and provides a structured foundation for subsequent pilot testing, policy integration, and phased implementation within Iran’s climate-vulnerable health system.

### International frameworks for climate-resilient health systems

Our Delphi-validated model should be interpreted within the broader landscape of global guidance on climate-resilient health systems. Frameworks such as the WHO Operational Framework for Climate-Resilient Health Systems outline core system functions needed to address climate-related health risks, including governance, resilient infrastructure, surveillance and early-warning systems, supply chains, financing, and workforce capacity^21,36,37^.

However, these frameworks are largely system-level and disease-agnostic, providing strategic direction rather than detailed guidance for the management of specific chronic conditions. In this context, the ICDMM can be understood as a disease-specific operationalization of broader climate–health principles for type 2 diabetes care. By translating general resilience domains into concrete actions for diabetes services—such as climate-sensitive surveillance, resilient insulin supply systems, and adaptive care delivery—the model provides a practical structure for integrating climate adaptation into routine diabetes management within Iran’s health system.

### Gaps in NCD/diabetes climate adaptation

The Delphi findings highlight a persistent gap in climate adaptation strategies for non-communicable diseases, particularly diabetes. Although the health impacts of climate change on diabetes—including heat exposure, environmental stressors, and disruptions to care—are increasingly documented, operational guidance for maintaining diabetes services during climate-related disruptions remains limited, particularly in low- and middle-income settings where climate vulnerability and diabetes burden often intersect^5,6^. Existing frameworks for climate-resilient health systems provide important strategic direction but generally remain disease-agnostic and do not specify how chronic disease services such as diabetes care should adapt within health systems^22^.

In addition, critical operational areas—including reliable access to medicines, resilient supply chains, and financing mechanisms to protect patients during climate-related shocks—are still insufficiently addressed in many adaptation strategies^38^. Global health authorities have increasingly emphasized that climate change and non-communicable diseases represent interconnected challenges requiring integrated responses within health systems^39^. The strong consensus achieved across the ICDMM domains suggests that these operational gaps are considered highly relevant by national experts. By translating broad climate–health principles into concrete actions for diabetes care—such as climate-sensitive surveillance, resilient insulin supply, adaptive financing, and continuity of care—the ICDMM helps bridge the divide between high-level climate resilience frameworks and the practical requirements of diabetes service delivery in a climate-vulnerable context.

### Innovations of the ICDMM

The Delphi process demonstrated strong and consistent endorsement for several components of the ICDMM that extend existing climate–health adaptation frameworks and align them specifically with the needs of diabetes care in Iran.

First, our Delphi panel strongly endorsed (83.3% agreement) the inclusion of psychosocial resilience as a core element of climate-responsive diabetes care, confirming its perceived importance for T2DM management in Iran. While climate–health literature increasingly recognises the mental-health impacts of climate stressors^37,40^, psychosocial support is rarely formalised within existing adaptation frameworks. WHO and Smart Buys acknowledge psychosocial stress following climate events^37,39^, but the ICDMM operationalises these concepts specifically for diabetes by integrating mental-health screening, counselling, peer support, and stress-coping interventions within routine care.

Second, panelists endorsed (94.4% agreement) the use of digital and AI-enabled surveillance systems to support early identification of climate-related risks for people with diabetes. In line with the broader paradigm of climate-smart public health^36^, the ICDMM integrates climate and health data within information systems and proposes the use of predictive analytics to anticipate climate-related surges in glycaemic events. Telemedicine and remote monitoring—already recommended in broader frameworks^37^—are adapted here into diabetes-specific protocols to maintain continuity of care during heatwaves or floods.

Third, the Delphi panel supported (88.9% agreement) the model’s emphasis on climate-sensitive financing mechanisms, viewing these as essential for protecting vulnerable people with diabetes during climate shocks. While global guidance highlights the need for innovative climate-health financing^38^, the ICDMM specifies actionable tools such as climate-indexed insurance, targeted subsidies for adaptive equipment, concessional loans during disasters, and monitoring of equity in out-of-pocket expenditures.

Fourth, community engagement and multisectoral collaboration were also judged important by the panel. (100% agreement after Round 1) ICDMM incorporates community health workers, NGOs, and volunteers in climate-related outreach and education. It also specifies mechanisms for intersectoral coordination—linking health, meteorology, agriculture, and education sectors—which aligns with global Health-in-All-Policies approaches^36,41^ but is operationalised here for diabetes care (e.g., heat-risk counselling, nutrition support during climate-related food disruptions).

Finally, the panel endorsed (94.4% agreement) strengthening supply-chain and infrastructure resilience, particularly for insulin and other temperature-sensitive commodities. The ICDMM emphasizes maintaining cold-chain stability, ensuring access to glucose-monitoring supplies, and integrating climate-adapted facility upgrades (e.g., solar backup power, low-emission cooling). While broader frameworks call for resilient health facilities^36^, the ICDMM calibrates these requirements specifically to diabetes service continuity.

Overall, the innovations of the ICDMM reflect expert consensus on a set of operational features—psychosocial support, digital surveillance, adaptive financing, community engagement, and supply-chain resilience—that are often underdeveloped or absent in existing climate-health frameworks but viewed as critical for delivering climate-responsive diabetes care in Iran.

### Comparative analysis and regional relevance

In light of the Delphi findings, the validated ICDMM both aligns with and extends existing models. Table 3 provides a direct comparison between the ICDMM and two key international frameworks – the WHO Operational Framework for Building Climate Resilient and Low Carbon Health Systems (2023) and the IDF climate change and diabetes recommendations – across the six WHO health system building blocks. As shown, while the WHO framework offers disease‑agnostic strategic guidance and the IDF provides general clinical recommendations, the ICDMM translates these into concrete, diabetes‑specific operational actions, including climate‑sensitive financing mechanisms, psychosocial support protocols, AI‑enabled surveillance, and equity‑focused workforce deployment.

**Table 3:** Comparison of ICDMM with selected existing frameworks.

WHO building block	WHO Operational Framework (disease-agnostic)	IDF recommendations (diabetes-specific but operational-light)	ICDMM (diabetes-specific + operational)
Leadership & governance	High-level guidance on climate-health governance	General calls for awareness	Climate-informed policies, equity oversight, cross-sectoral coordination
Service delivery	Resilient infrastructure, essential services	Heat-related advice for patients	Psychosocial resilience, continuity protocols during extreme events, community-based outreach
Health workforce	Training, surge capacity	Acknowledges need for clinician awareness	Competency-based climate curricula, deployment to high-risk areas, mental health support for staff
Health information systems	Early warning, climate data integration	None specified	AI-enabled predictive analytics, diabetes-specific surveillance, telemedicine protocols
Medical products & technologies	Supply chain resilience, cold chain	Mentions insulin storage	Diabetes-specific cold chain, diversified suppliers, low-emission backup power
Health financing	Calls for innovative financing	None specified	Climate-indexed insurance, targeted subsidies, equity-monitoring mechanisms

Like WHO’s framework and CSPH, it advocates for resilient infrastructure, early warning, surveillance and integration of climate data^21,36^. It similarly calls for trained, flexible health workforces and multisectoral governance. Where it diverges is in its disease-specific depth and scope: it explicitly tailors every building block to diabetes, addressing psychosocial support, nutrition, and finance in ways often glossed over by broader plans. This reflects ICDMM’s origin: a mixed-methods study in Iran identified local climate triggers (e.g. sandstorms causing pollution spikes) and health system gaps (e.g. insulin stockouts) that generic guidance had missed.

We acknowledge that some high-level components (e.g., governance, workforce training) could apply to other NCDs. However, the ICDMM includes several diabetes-specific elements that would not automatically transfer: insulin cold-chain protocols, hypoglycaemia prevention during heatwaves, continuous glucose monitoring adaptations, and diabetes-tailored nutritional support during food insecurity. These features make the model disease-specific while still allowing cross-learning for other climate-sensitive conditions.

Global gaps in NCD adaptation are exactly what ICDMM aims to fill. WHO notes that climate and NCD strategies “should be aligned in synergistic interventions”^39^, but few national plans do so comprehensively. In the UK and Australia, for example, climate adaptation reports largely focus on heat emergency preparedness and infrastructure, with limited mention of chronic care continuity. ICDMM’s approach could guide Low-and middle-income countries (LMICs) in South Asia and the Middle East, where climate stressors and diabetes burdens co-exist. Its emphasis on AI-driven warnings and digital care reflects lessons from climate-health pilots in Madagascar and elsewhere^36^, while its finance and equity components address issues highlighted by WHO and World Bank analyses^22,38^.

In diverse contexts (LMICs, Australia, Canada, South Asia), health adaptation plans vary: some stress heat action plans or vector control, others emphasize sustainable diets and exercise. ICDMM complements these by adding clinically focused measures. For example, unlike many Western plans, ICDMM explicitly incorporates culturally sensitive dietary support under climate stress (food insecurity), which is crucial in countries facing both malnutrition and diabetes. The model’s community-based outreach and psychosocial resilience also resonate with Disaster Risk Reduction frameworks in Asia-Pacific that call for local empowerment and mental health first aid during disasters.

Overall, the ICDMM provides a context-specific operational framework that translates broad climate–health principles into practical strategies for diabetes care. It integrates operational elements—including psychosocial support, AI-assisted surveillance, and climate-sensitive financing—into a coordinated framework that may inform adaptation efforts in other settings. While WHO and Lancet-driven frameworks provide the architecture for climate-resilient health systems^21,36^, the ICDMM adds specificity: it operationalizes these principles for diabetes management in a high-risk setting. This may help address some of the operational gaps identified in current climate adaptation efforts for chronic disease care^5,22^ and illustrates how to translate broad climate-health guidance into concrete chronic disease policies.

### Strengths and limitations

This study’s strengths include its rigorous Delphi methodology and multidisciplinary expertise. The two-round consensus process, guided by established protocols (ACCORD guidelines), ensured systematic convergence of expert opinions. Anonymous, iterative feedback allowed panellists from diverse backgrounds (endocrinology, primary care, disaster preparedness, health policy, etc.) to refine the model without dominance bias. The panel, though modest in size (18 experts), represented multiple disciplines and levels of the health system, which bolsters the credibility of the findings. Achieving consensus on all components is uncommon and indicates broad support for the model’s relevance within this expert group, while still warranting further external validation.

However, some limitations merit caution. Consensus does not guarantee optimal efficacy: expert agreement reflects perceived validity, not tested implementation or real-world effectiveness. dichotomous “agree/disagree” format was chosen to provide clarity and minimise respondent burden – strengths that likely contributed to the 100% retention rate and complete data. However, this binary scale has important trade‑offs. First, it forced panellists to reduce potentially nuanced judgements into a simple agree/disagree dichotomy, making it impossible to distinguish between components that are “essential” versus those that are merely “desirable” or “helpful”. Second, by not allowing intermediate responses (such as “partially agree” or “neutral”), the scale may have artificially inflated the apparent level of consensus, because panellists who had minor reservations were still counted as agreeing. Third, the binary format prevents assessment of the relative importance or feasibility of each component – information that would be valuable for prioritising implementation. Future studies should complement this binary validation with Likert‑type ratings (e.g., 1–9 importance scales) to capture gradations of expert opinion and to identify which components are both strongly endorsed and practically feasible. Readers should interpret the high consensus percentages with these methodological trade‑offs in mind.

Furthermore, because the Delphi statements were formulated as general principles rather than highly granular, context‑specific actions, the high consensus levels may partly reflect agreement with common norms rather than validated operational detail. We have therefore supplemented the model with an operationalisation framework (Supplementary Table S3) that provides concrete, implementable steps for key components.

In addition to this methodological constraint, the composition of the panel also shaped the scope of the validation. The panel’s composition, while diverse, was predominantly academic and clinical; other perspectives (e.g. patients, community health workers) were not included, and no international experts participated. This absence is a notable gap, particularly for components related to psychosocial resilience, financial protection, and service delivery, where lived experience, frontline realities, and cross-context perspectives are essential for assessing feasibility and acceptability. Sample size was relatively small by survey standards, which may limit generalizability even within Iran. Nonetheless, a panel of 18 experts falls within the commonly recommended range for Delphi studies (typically 10–30 participants) for focused content validation in a single‑country context, and the 100% retention rate between rounds strengthens confidence in the stability of consensus (Hsu & Sandford, 2007; Keeney et al., 2011). Moreover, the Delphi threshold (≥ 70%) is conventional but arbitrary; items reaching minimal consensus in round 1 had to be reworded, indicating initial ambiguity in some statements.

Finally, the study’s focus on Iran means caution when extrapolating to other countries, particularly because no international experts were included in the Delphi panel. Contextual factors (political structure, culture, resource levels) may differ, so some ICDMM elements might require adaptation elsewhere. Furthermore, the same research team that designed the ICDMM also developed and administered the Delphi questionnaires, which may introduce confirmation bias despite efforts to incorporate open-ended feedback and pre-specified thresholds. Future validation exercises led by independent teams, and involving additional stakeholder groups such as patients, community health workers and payers, would help to mitigate this risk and test the model’s transferability.

### Implications for policy and practice

For Iranian health policymakers and practitioners, the validated ICDMM offers a clear, actionable framework to integrate climate resilience into diabetes care. Each domain maps to policy levers: Governance efforts could institutionalize climate-smart diabetes policies and cross-ministry coordination; Workforce planning can prioritize deployment and training of diabetes specialists in drought- or heat-prone provinces; Service Delivery initiatives might develop patient education on heat management, empower communities for psychosocial support, and ensure continuity of care during floods or power outages; Information Systems enhancements could include syndromic surveillance for heat-related diabetes events and use climate data for forecasting service needs; Medical Products strategies involve securing cold chains for insulin, diversifying suppliers, and promoting low-carbon facility design; Financing reforms could expand insurance coverage and rapid assistance for diabetics after disasters. Together, these actions align with WHO’s recommendations for resilient health systems (integrating equity, sustainability and preparedness)^39,42^.

In practice, the ICDMM can guide the Ministry of Health to revise national diabetes guidelines and emergency protocols, and to incorporate climate risk into existing NCD programs. Training curricula for health workers can include climate-health modules, as the model emphasizes climate–health knowledge. Provincial health authorities in vulnerable regions (e.g. Khuzestan, Sistan) can use the ICDMM to audit their readiness and prioritize investments (e.g. emergency stockpiles of medicines, telemedicine capacity). Importantly, the model underscores equity – for example, climate-sensitive insurance packages and monitoring of financial access – which may help protect the poorest diabetics during crises. These implications extend to similar LMIC settings: any climate-vulnerable country with a sizable diabetes burden can adapt the ICDMM’s domains to its context.

To provide the level of operational detail expected for diabetes clinics, primary care teams, and provincial health authorities, Supplementary Table S3 offers a concrete operationalisation of ten key ICDMM components. For each component, the table specifies: responsible actors at national, provincial, and facility levels; step-by-step implementation actions with indicative timelines (e.g., 3 months, 6 months, 1 year); and measurable process indicators (e.g., percentage of clinics with screening protocols, number of trained health workers, supply chain restoration time). This turns the conceptual framework into an actionable guide for local implementation.

### Future research directions

Building on this validation, future work should focus on implementation and evaluation. Pilot studies are needed to test ICDMM-guided interventions (e.g. establishing a climate-aware diabetes registry or integrating heat warnings into diabetes clinics) and to assess their impact on outcomes (hospitalizations, glycemic control, patient well-being). Research should measure cost-effectiveness of recommended measures (e.g. community-based cooling centers or telehealth) in Iran’s resource context. Comparative studies could adapt the ICDMM to other LMICs’ contexts (e.g. in the Middle East or South Asia) to evaluate generalizability and to identify context-specific modifications. Further stakeholder engagement, including patients and frontline providers, can refine the model and inform culturally appropriate implementation. Policy analysis is also warranted: for example, examining how Iran’s health and climate plans can integrate the ICDMM elements, or assessing barriers to intersectoral action as suggested by WHO.

Ultimately, evidence must be generated on whether climate-smart diabetes management (as structured by the ICDMM) improves resilience. This aligns with recent calls for evidence-based adaptation: mapping and evaluating health system responses globally can identify best practices and gaps. In line with the Lancet Countdown and WHO’s health adaptation goals, longitudinal studies should track whether applying the ICDMM reduces climate-related diabetes morbidity and mortality over time. By testing and refining the model through implementation research, future studies in Iran may generate valuable evidence for operationalizing climate-resilient NCD care in comparable LMIC settings.

## Conclusion

This Delphi study supports the content validity of the Integrated Iranian Climate-Sensitive Diabetes Management Model (ICDMM) as a health system–oriented framework for addressing type 2 diabetes under climate-related stress in Iran. Through a structured two-round consensus process with a multidisciplinary panel of national experts, all 51 statements mapped to the six WHO health system building blocks reached the predefined consensus threshold, indicating strong expert agreement on the model’s perceived relevance, coherence, and completeness within the Iranian context. The ICDMM translates high-level global guidance on climate-resilient and low-carbon health systems into a structured, disease-specific, and context-sensitive framework for diabetes care in Iran. It explicitly incorporates key dimensions such as psychosocial resilience, digital and AI-enabled information systems, resilient supply chains, and climate-sensitive financing for people living with diabetes. In this way, it provides an expert-informed basis for integrating climate resilience into diabetes care, while remaining a consensus-based framework rather than an implementation-tested intervention.

At the same time, expert consensus represents an important but preliminary step in model development. The ICDMM now requires rigorous implementation and evaluation in real-world settings to determine its feasibility, effectiveness, affordability and equity impacts, particularly in highly exposed provinces. Pilot programmes, participatory adaptation with patients and frontline providers, and comparative studies in other low- and middle-income countries are needed to refine the model and test its transferability. By moving from concept to practice, the ICDMM may help inform efforts to strengthen Iran’s readiness for the intersecting crises of diabetes and climate change, and may offer lessons for similar climate-vulnerable settings, pending further implementation research. While the ICDMM achieved strong expert agreement on its relevance, coherence, and completeness, this Delphi study establishes content validity and consensus rather than real-world effectiveness. The model now requires pilot implementation and evaluation in routine practice to determine its feasibility, operational requirements, and impact on diabetes care under climate-related stressors.

## Supplementary Information

Below is the link to the electronic supplementary material.

Supplementary Material 1

Supplementary Material 2

Supplementary Material 3

Supplementary Material 4

## Data Availability

The datasets generated and/or analyzed during the current study are available from the corresponding author upon reasonable request.

## Abbreviations

Abbreviation: Full term
ACCORD: Accurate Consensus Reporting Document
AI: Artificial intelligence
BCP: Business continuity plan
BSc: Bachelor of Science
CSPH: Climate-smart public health
HIS: Health information system
ICDMM: Integrated Iranian Climate-Sensitive Diabetes Management Model
IDF: International Diabetes Federation
LMICs: Low- and middle-income countries
MSc: Master of Science
NCD: Noncommunicable disease
NGO: Non-governmental organization
SD: Standard deviation
SOP: Standard operating procedure
SPSS: Statistical Package for the Social Sciences
T2DM: Type 2 diabetes mellitus
UNDRR: United Nations Office for Disaster Risk Reduction
