# Supplementary material for: Delphi consensus validation of an integrated climate sensitive diabetes management model for climate vulnerable health systems in Iran
Source: Sci Rep. 2026 Jun 8;16:25943. doi: 10.1038/s41598-026-56784-z (PMC13487174; doi:10.1038/s41598-026-56784-z)
Supplement: Supplementary file 1 [file 41598_2026_56784_MOESM1_ESM.docx]

# Additional file 1: ACCORD Checklist for "Validating the Integrated Iranian Climate-Sensitive Diabetes Management Model (ICDMM): a Delphi consensus study"

This table summarises how the manuscript reports key elements of the ACCORD (ACcurate COnsensus Reporting Document) guideline. Locations are referenced by manuscript section and content; page numbers may vary depending on journal formatting, so they should be adjusted if needed in the final version.

| ACCORD item code | Manuscript section | Reporting recommendation (paraphrased) | Reported? | Location in this manuscript |
| --- | --- | --- | --- | --- |
| T1 | Title | State that the article reports a consensus exercise and name the consensus method in the title. | Yes | Title page – main title: “Validating the Integrated Iranian Climate-Sensitive Diabetes Management Model (ICDMM): a Delphi consensus study”. (page 1) |
| I1 | Introduction | Explain why a consensus approach (e.g. Delphi) was chosen instead of other designs. | Yes | Introduction – final paragraphs and Methods – Study design (rationale for Delphi to refine and validate the ICDMM). (page 4) |
| I2 | Introduction | State the aims of the consensus exercise and, where relevant, its target audience and geographical scope. | Yes | Introduction – final two paragraphs (aim of validating ICDMM for Iran; focus on national health system and climate‑vulnerable provinces). (page 4) |
| I3 | Introduction | If the exercise updates or builds on previous consensus work, describe this and provide references; otherwise, make clear it is a new exercise. | Yes | Introduction – final paragraph (describes this as the first Delphi‑validated, climate‑sensitive, health‑system model for T2DM in a middle‑income country; no previous consensus to update). (page 4) |
| M1 | Methods – Study design | Indicate whether a protocol for the consensus exercise was prospectively registered and, if so, where. | yes | The Delphi protocol was not prospectively registered. Page 5 |
| M2 | Methods – Study design / Authors’ contributions | Describe who led and coordinated the consensus exercise and their roles (e.g. steering group, methodologists, content experts). | Yes | Methods – Study design (research team identified) and Declarations – Authors’ contributions (roles in design, supervision and analysis). (pages 5,20) |
| M3 | Methods – Participants | Specify eligibility criteria for panellists and justify key criteria (e.g. expertise, years of experience). | Yes | Methods – Participants (explicit criteria: ≥5 years relevant experience, relevant degree, willingness to join both rounds).(page 6-7) |
| M4 | Methods – Participants | Describe how panellists were identified and recruited, including sampling approach and invitations. | Yes | Methods – Participants (purposive sampling via national/provincial NCD and diabetes committees, MOHME working groups, academic networks, plus snowballing). (pages 6-7) |
| M5 | Methods – Participants / Results – Participant characteristics | Provide information on panel composition and characteristics (e.g. disciplines, sectors, geography). | Yes | Methods – Participants (disciplines and institutional affiliations) and Results – Participant characteristics with Table 1 (age, gender, profession, experience, fields of expertise). (pages 6-7) |
| M6 | Methods – Development of the Delphi questionnaire | Describe the sources of information used to generate items and any evidence provided to panellists (e.g. literature reviews, qualitative studies). | Yes | Methods – Development of the Delphi questionnaire (scoping review and qualitative study as sources; mapping to WHO building blocks). (page 5-6) |
| M7 | Methods – Development of the Delphi questionnaire / Supplementary material | Describe how initial items/statements were developed, refined and organised before the first round. | Yes | Methods – Development of the Delphi questionnaire (iterative team review; merging overlaps; 51 statements in six domains) and Supplementary Table S1 (mapping matrix). (pages 5-6) |
| M8 | Methods – Study design | Name and briefly describe the consensus technique(s) used and any modifications made (e.g. modified two‑round Delphi). | Yes | Methods – Study design (two‑round, cross‑sectional Delphi consensus exercise; rationale for using Delphi; reference to ACCORD). Page 5 |
| M9 | Methods – Data collection and analysis | Specify the number of rounds/steps planned, stopping rules, and the definition of consensus (threshold and metrics). | Yes | Methods – Data collection and analysis (maximum of two rounds pre‑specified, option of a third; consensus threshold ≥70% agreement; categories of strong/moderate/low consensus; no third round required). Page 6-7 |
| M10 | Methods – Data collection and analysis | Describe how feedback between rounds was provided to panellists. | Yes | Methods – Data collection and analysis (second‑round questionnaire sent with summary of Round‑1 agreement for all items; panellists invited to reconsider responses). Page 6-7 |
| M11 | Methods – Study design | Describe whether and how responses were anonymous during the consensus process. | yes | Methods – Data collection and analysis (para 1: description of unique study IDs, de-identified questionnaires and aggregate group-level feedback ensuring anonymity).Page 6 |
| M12 | Methods – Data collection and analysis | Describe how quantitative data (e.g. ratings, votes) were collected and analysed. | Yes | Methods – Data collection and analysis (binary agree/disagree scale; descriptive statistics with SPSS; thresholds and categorisation of consensus). Page 7-8 |
| M13 | Methods – Data collection and analysis | Describe how qualitative data (e.g. comments) were collected, analysed and used to modify items. | Yes | Methods – Data collection and analysis (free‑text comments exported to Excel; independent review by two researchers; thematic grouping; disagreements resolved with a third senior researcher; used to reword ambiguous items). Page 7-8 |
| M14 | Methods – Data collection and analysis | Describe how items were added, removed or modified during the exercise and any protocol deviations. | Yes | Methods – Data collection and analysis (five statements with low agreement or ambiguity revised after Round 1; explicit statement that no major deviations from the pre‑specified protocol occurred). Page 7-8 |
| M15 | Methods – Participants / Data collection and analysis / Results – Participant characteristics | Report how non‑response, attrition and missing data were handled. | Yes | Methods – Participants (30 invited, 18 responded and entered Round 1, all 18 completed Round 2); Methods – Data collection and analysis (only complete questionnaires included, no missing data; 100% retention between rounds). Pages 5-6 |
| R1 | Methods – Data collection and analysis | State when the consensus exercise took place and the timing/duration of each round. | Yes | Methods – Data collection and analysis (Delphi rounds conducted between September 2025 and November 2025; each round open for ~2 weeks with scheduled reminders). Page 6 |
| R2 | Results – Participant characteristics | Report the flow of panellists through the exercise (numbers invited, responded, and analysed at each round). | Yes | Methods – Participants (30 invited; 18 completed Round 1 and Round 2) and Results – Participant characteristics (N=18 included in analysis). Page 9 |
| R3 | Results – Delphi consensus process / Table 2 | Present the main quantitative consensus results for each item (e.g. agreement percentages per round). | Yes | Results – Delphi consensus process and Table 2 (agreement range per round, number of statements reaching consensus, and final status for all 51 items). Pages 9-11 |
| R4 | Results – Delphi consensus process | Summarise key themes from qualitative feedback and how they influenced revisions to items. | Yes | Results – Delphi consensus process (narrative summary of main concerns about psychosocial resilience, workforce distribution, supply chains, financial protection and equity; description of how comments guided rewording). Page 9-11 |
| D1 | Discussion – Strengths and Limitations | Discuss methodological strengths and limitations of the consensus exercise (e.g. panel composition, response rates, anonymity, potential biases). | Yes | Discussion – Strengths and Limitations (details on sample size, expert mix, binary scale, lack of patient involvement, potential confirmation bias, generalisability). Page 16-17 |
| D2 | Discussion – International Frameworks / Gaps / Innovations / Comparative Analysis | Interpret the findings in relation to existing evidence, frameworks and similar consensus exercises. | Yes | Discussion – International Frameworks for Climate‑Resilient Health Systems; Gaps in NCD/Diabetes Climate Adaptation; Innovations of the ICDMM; Comparative Analysis and Regional Relevance. Pages 13-15 |
| D3 | Discussion – Implications / Future Research / Conclusion | Discuss implications for policy, practice and future research, and how the recommendations should be used. | Yes | Discussion – Implications for Policy and Practice; Future Research Directions; Conclusion (use of ICDMM in Iranian health policy and potential adaptation to other LMICs). Pages 17-19 |
| O1 | Declarations – (not applicable) | List any organisations that endorsed the consensus recommendations and describe their role. | No / Not applicable | No endorsing professional body or organisation is reported; the work is presented as an academic research study rather than a guideline formally endorsed by an external organisation. |
| O2 | Declarations – Funding / Competing interests | Report funding sources, their role in the work, and any competing interests. | Yes | Declarations – Funding (no specific grant; funders had no role) and Competing interests (authors declare no competing interests). Page 20 |
| O3 | Declarations – Availability of data and material / Supplementary material | State whether data, materials and supplementary files (e.g. item lists, questionnaires) are available and how they can be accessed. | Yes | Declarations – Availability of data and material (datasets available on reasonable request) and reference to Supplementary Table S1 (matrix linking scoping review, qualitative themes and Delphi items). Page 20 |
