# Supplementary material for: Delphi consensus validation of an integrated climate sensitive diabetes management model for climate vulnerable health systems in Iran
Source: Sci Rep. 2026 Jun 8;16:25943. doi: 10.1038/s41598-026-56784-z (PMC13487174; doi:10.1038/s41598-026-56784-z)
Supplement: Supplementary file 2 [file 41598_2026_56784_MOESM2_ESM.docx]

**Supplementary Table S1. Mapping of WHO health system building blocks, scoping-review domains, qualitative themes and final ICDMM components used to generate the Delphi statements**

| **WHO building block** | **Scoping-review domain** | **Qualitative theme** | **Final ICDMM component (ICDMM)** | **Delphi item code** |
| --- | --- | --- | --- | --- |
| Leadership and governance | Integrated policy, governance and planning | Integrated governance and forward-looking health policy | Forward-looking, climate-informed policy-making with continuous review | LG1 |
|  |  |  | Integrated governance and intersectoral collaboration with strong political commitment | LG2 |
|  |  |  | Evidence-informed and locally responsive policy-making | LG3 |
|  |  |  | Transparent systems for monitoring, evaluation and accountability with enforceable mechanisms | LG4 |
|  |  |  | Development and regular updating of climate- and crisis-oriented Standard operating procedures (SOPs) / Business continuity plan (BCPs) | LG5 |
|  |  |  | Scientific collaboration and international exchange of experience | LG6 |
|  |  |  | Adoption of equity-oriented policies and protection of vulnerable groups | LG7 |
|  |  |  | Strengthening legal frameworks and enforceable regulations in the climate–health domain | LG8 |
| Service delivery | Health infrastructure and operations | Smart infrastructure and operational resilience of the health system | Establishing climate-resilient infrastructure to ensure continuity of services during crises | SD1 |
|  |  |  | Operational preparedness and rapid response of facilities and primary care networks | SD2 |
|  | Community-based education, empowerment and behaviour change | Empowerment of human capital and community-based behaviour change | Education and empowerment of patients and the community | SD3 |
|  |  |  | Social participation and mobilization of Non-governmental organizations (NGOs) for prevention | SD4 |
|  | Social, economic and environmental determinants | Financial equity and health-system sustainability in the context of social, economic and environmental determinants | Provision of psychosocial support for patients and their families | SD5 |
|  |  |  | Reducing inequities and improving access to essential health services | SD6 |
|  | Technology, data analytics, early warning systems and digital health | Digital transformation, data-driven approaches and early warning systems for health | Expansion of smart services (telemedicine and electronic health records) | SD7 |
|  | Mental health and psychosocial impacts | Empowerment of human capital and community-based behaviour change | Strengthening psychosocial resilience of people with type 2 diabetes and their families in the face of climate-related crises | SD8 |
|  |  |  | Designing climate-sensitive nutritional and food-support programmes for patients during crises | SD9 |
| Health workforce | Community-based education, empowerment and behaviour change | Empowerment of human capital and community-based behaviour change | Fair distribution of, and support for, specialised diabetes workforce in climate high-risk areas | HW1 |
|  |  |  | Training on climate–health interactions and crisis management | HW2 |
|  |  |  | Strengthening screening and clinical decision-making skills during crises | HW3 |
|  |  |  | Continuous professional development and updating the knowledge of health workers | HW4 |
|  |  |  | Psychological support and occupational safety for staff during crises | HW5 |
|  |  |  | Risk-based workforce planning and replacement during emergencies | HW6 |
|  | Mental health and psychosocial impacts |  | Scientific networking to strengthen education and research | HW7 |
|  |  |  | Development of supportive and counselling programmes for staff mental health in climate-related crises | HW8 |
| Health information systems | Technology, data analytics, early warning systems and digital health | Digital transformation, data-driven approaches and early warning systems for health | Early-warning and public alert systems | HIS1 |
|  |  |  | Targeted messaging to patients during crises | HIS2 |
|  |  |  | Digital education and continuity of remote care | HIS3 |
|  |  |  | Improving the quality and interoperability of health data | HIS4 |
|  |  |  | Data analysis and decision-support tools for policy-making | HIS5 |
|  |  |  | Integration of climate data into the health information system (HIS) | HIS6 |
|  |  |  | Resilience of information and communication infrastructure during crises | HIS7 |
|  |  |  | Improved recording and analysis of climate-related health data | HIS8 |
|  |  |  | Use of artificial intelligence and data mining to predict climate-related crises | HIS9 |
| Medical products and technologies | Health infrastructure and operations | Smart infrastructure and operational resilience of the health system | Resilient supply chain for medicines and medical equipment | MPT1 |
|  |  |  | Managing supply bottlenecks in vulnerable and hard-to-reach areas | MPT2 |
|  |  |  | Expansion of digital health and information technologies | MPT3 |
|  |  |  | Establishment of green hospitals and low-carbon health-care systems | MPT4 |
|  |  |  | Waste management and promotion of environmental health in health-care facilities | MPT5 |
|  |  |  | Technological innovation (remote monitoring, smart tools and devices) | MPT6 |
|  | Digital transformation, data-driven approaches and early warning systems for health |  | Resilient supply of critical medicines and equipment during crises | MPT7 |
|  |  |  | Development of new technologies for remote monitoring of patients | MPT8 |
|  |  |  | Stable and reliable supply of diabetes medicines and devices during climate-related crises | MPT9 |
| Health financing | Social, economic and environmental determinants | Financial equity and health-system sustainability in the context of social, economic and environmental determinants | Adequate insurance coverage and financial protection for people with type 2 diabetes in climate high-risk situations | HF1 |
|  |  |  | Financial support to prevent interruption of diabetes care and treatment | HF2 |
|  |  |  | Establishing flexible and sustainable financial resources for crisis preparedness and response | HF3 |
|  |  |  | Reducing insurance gaps and financial deprivation among patients | HF4 |
|  |  |  | Improving the efficiency of health expenditures and reforming supportive financing policies | HF5 |
|  |  |  | Financial innovations (results-based schemes, vouchers and health loans) | HF6 |
|  |  |  | Monitoring equity in payments for type 2 diabetes care and improving access and continuity of services | HF7 |
|  |  |  | Designing climate-sensitive insurance packages for patients in high-risk conditions | HF8 |
