# Supplementary material for: Delphi consensus validation of an integrated climate sensitive diabetes management model for climate vulnerable health systems in Iran
Source: Sci Rep. 2026 Jun 8;16:25943. doi: 10.1038/s41598-026-56784-z (PMC13487174; doi:10.1038/s41598-026-56784-z)
Supplement: Supplementary file 3 [file 41598_2026_56784_MOESM3_ESM.docx]

| Supplementary Table S2: Final Delphi Results for the Climate-Sensitive Type 2 Diabetes Management Model | | | | | | | |
| --- | --- | --- | --- | --- | --- | --- | --- |
| Model Layer | **Domain / Section** | **Statement / Item** | **Total Respondents (N)** | **Agreement in Round 1 (%)** | **Agreement in Round 2 (%)** | **Final Status** |  |
| Health System Building Blocks | **Leadership & Governance** | Forward-looking, climate-informed policy-making with continuous review | 18 | 88.9 | — | Final approval |  |
|  |  | Integrated governance and intersectoral collaboration with strong political commitment | 18 | 100 | — | Final approval |  |
|  |  | Evidence-informed and locally responsive policy-making | 18 | 100 | — | Final approval |  |
|  |  | Transparent systems for monitoring, evaluation and accountability with enforceable mechanisms | 18 | 100 | — | Final approval |  |
|  |  | Development and regular updating of climate- and crisis-oriented Standard operating procedures (SOPs) / Business continuity plan (BCPs) | 18 | 88.9 | — | Final approval |  |
|  |  | Scientific collaboration and international exchange of experience | 18 | 94.4 | — | Final approval |  |
|  |  | Adoption of equity-oriented policies and protection of vulnerable groups | 18 | 100 | — | Final approval |  |
|  |  | Strengthening legal frameworks and enforceable regulations in the climate–health domain | 18 | 83.3 | — | Final approval |  |
|  | **Service Delivery** | Establishing climate-resilient infrastructure to ensure continuity of services during crises | 18 | 100 | — | Final approval |  |
|  |  | Operational preparedness and rapid response of facilities and primary care networks | 18 | 100 | — | Final approval |  |
|  |  | Education and empowerment of patients and the community | 18 | 94.4 | — | Final approval |  |
|  |  | Social participation and mobilization of Non-governmental organizations (NGOs) for prevention | 18 | 88.9 | — | Final approval |  |
|  |  | Provision of psychosocial support for patients and their families | 18 | 88.9 | — | Final approval |  |
|  |  | Reducing inequities and improving access to essential health services | 18 | 100 | — | Final approval |  |
|  |  | Expansion of smart services (telemedicine and electronic health records) | 18 | 88.9 | — | Final approval |  |
|  |  | Strengthening psychosocial resilience of people with type 2 diabetes and their families in the face of climate-related crises | 18 | 66.7 | 83.3 | Final approval after revision (Round 2) |  |
|  |  | Designing climate-sensitive nutritional and food-support programmes for patients during crises | 18 | 83.3 | — | Final approval |  |
|  | **Health Workforce** | Fair distribution of, and support for, specialised diabetes workforce in climate high-risk areas | 18 | 61.1 | 88.9 | Final approval after revision (Round 2) |  |
|  |  | Training on climate–health interactions and crisis management | 18 | 100 | — | Final approval |  |
|  |  | Strengthening screening and clinical decision-making skills during crises | 18 | 100 | — | Final approval |  |
|  |  | Continuous professional development and updating the knowledge of health workers | 18 | 94.4 | — | Final approval |  |
|  |  | Psychological support and occupational safety for staff during crises | 18 | 88.9 | — | Final approval |  |
|  |  | Risk-based workforce planning and replacement during emergencies | 18 | 88.9 | — | Final approval |  |
|  |  | Scientific networking to strengthen education and research | 18 | 77.8 | — | Final approval |  |
|  |  | Development of supportive and counselling programmes for staff mental health in climate-related crises | 18 | 83.3 | — | Final approval |  |
|  | **Health Information Systems** | Early-warning and public alert systems | 18 | 100 | — | Final approval |  |
|  |  | Targeted messaging to patients during crises | 18 | 100 | — | Final approval |  |
|  |  | Digital education and continuity of remote care | 18 | 100 | — | Final approval |  |
|  |  | Improving the quality and interoperability of health data | 18 | 100 | — | Final approval |  |
|  |  | Data analysis and decision-support tools for policy-making | 18 | 100 | — | Final approval |  |
|  |  | Integration of climate data into the health information system (HIS) | 18 | 100 | — | Final approval |  |
|  |  | Resilience of information and communication infrastructure during crises | 18 | 100 | — | Final approval |  |
|  |  | Improved recording and analysis of climate-related health data | 18 | 100 | — | Final approval |  |
|  |  | Use of artificial intelligence and data mining to predict climate-related crises | 18 | 94.4 | — | Final approval |  |
|  | **Medical Products, Vaccines & Technologies** | Resilient supply chain for medicines and medical equipment | 18 | 100 | — | Final approval |  |
|  |  | Managing supply bottlenecks in vulnerable and hard-to-reach areas | 18 | 100 | — | Final approval |  |
|  |  | Expansion of digital health and information technologies | 18 | 100 | — | Final approval |  |
|  |  | Establishment of green hospitals and low-carbon health-care systems | 18 | 72.2 | — | Final approval |  |
|  |  | Waste management and promotion of environmental health in health-care facilities | 18 | 83.3 | — | Final approval |  |
|  |  | Technological innovation (remote monitoring, smart tools and devices) | 18 | 100 | — | Final approval |  |
|  |  | Resilient supply of critical medicines and equipment during crises | 18 | 88.9 | — | Final approval |  |
|  |  | Development of new technologies for remote monitoring of patients | 18 | 100 | — | Final approval |  |
|  |  | Stable and reliable supply of diabetes medicines and devices during climate-related crises | 18 | 66.7 | 94.4 | Final approval after revision (Round 2) |  |
|  | **Health Financing** | Adequate insurance coverage and financial protection for people with type 2 diabetes in climate high-risk situations | 18 | 66.7 | 88.9 | Final approval after revision (Round 2) |  |
|  |  | Financial support to prevent interruption of diabetes care and treatment | 18 | 100 | — | Final approval |  |
|  |  | Establishing flexible and sustainable financial resources for crisis preparedness and response | 18 | 100 | — | Final approval |  |
|  |  | Reducing insurance gaps and financial deprivation among patients | 18 | 88.9 | — | Final approval |  |
|  |  | Improving the efficiency of health expenditures and reforming supportive financing policies | 18 | 94.4 | — | Final approval |  |
|  |  | Financial innovations (results-based schemes, vouchers and health loans) | 18 | 83.3 | — | Final approval |  |
|  |  | Monitoring equity in payments for type 2 diabetes care and improving access and continuity of services | 18 | 61.1 | 83.3 | Final approval after revision (Round 2) |  |
|  |  | Designing climate-sensitive insurance packages for patients in high-risk conditions | 18 | 83.3 | — | Final approval |  |
