# Supplementary material for: Delphi consensus validation of an integrated climate sensitive diabetes management model for climate vulnerable health systems in Iran
Source: Sci Rep. 2026 Jun 8;16:25943. doi: 10.1038/s41598-026-56784-z (PMC13487174; doi:10.1038/s41598-026-56784-z)
Supplement: Supplementary file 4 [file 41598_2026_56784_MOESM4_ESM.docx]

**Supplementary Table S3 – Operationalisation of key ICDMM components: responsible actors, initial implementation steps, and process indicators**

| Contentious items (revised after Round 1) | Statement (from ICDMM) | Domain | Final agreement (%) | Responsible actor(s) | Initial implementation steps | Process indicators |
| --- | --- | --- | --- | --- | --- | --- |
| 1 | Strengthening psychosocial resilience of people with type 2 diabetes and their families in the face of climate-related crises | Service Delivery | 83.30% | Deputy for Health (MOHME), Mental Health Department, Medical Universities of high-risk provinces (Khuzestan, Sistan‑Baluchestan, Hormozgan) | 1. Within 3 months, develop a screening protocol for mental health (anxiety, depression, PTSD) for diabetes patients exposed to climate stress. | Percentage of diabetes clinics with the screening protocol (target: 50% in 1 year). |
|  |  |  |  |  | 2. Conduct a 2‑day training workshop for community health workers (behvarzes) to provide peer support and psychological first aid. | Number of counselling sessions provided to patients and families during a crisis. |
|  |  |  |  |  | 3. Establish a telephone counselling helpline for diabetes patients during climate crises (heatwaves, severe dust storms). | Mean GHQ‑12 score change pre‑/post‑intervention. |
| 2 | Fair distribution of, and support for, specialised diabetes workforce in climate high-risk areas | Health Workforce | 88.90% | Deputy for Treatment (MOHME), Deputy for Management Development (MOHME), Medical Council Organisation | 1. Within 6 months, revise workforce allocation formula by adding a “climate vulnerability index” (weighting provinces with extreme heat, dust storms, drought). | Ratio of endocrinologists/diabetes specialists to diabetes population in high‑risk provinces vs. national average (target: reduce gap to <20% within 3 years). |
|  |  |  |  |  | 2. Design a support package: climate‑specific salary supplement, housing facilities, tele‑education, and tele‑endocrinology backup for specialists in high‑risk areas. | Annual staff satisfaction and retention survey scores. |
|  |  |  |  |  | 3. Pilot a “diabetes family physician” scheme in two provinces (Khuzestan and Sistan‑Baluchestan) recruiting at least 50 new nutritionists and 20 endocrinologists. | Number of tele‑endocrinology consultations performed. |
| 3 | Stable and reliable supply of diabetes medicines and devices during climate-related crises | Medical Products, Vaccines & Technologies | 94.40% | Iran Food and Drug Administration, Regional medical warehouses, Provincial Disaster Management Offices | 1. Within 3 months, map cold chain vulnerabilities for insulin and injectable diabetes medicines (power cuts, floods, dust storms). | Number of days with zero medicine stockouts during a crisis (target: 0 days). |
|  |  |  |  |  | 2. Establish a 30‑day emergency stockpile of essential medicines (insulin, metformin, glibenclamide) and devices (glucose test strips) in high‑risk province warehouses, replenished monthly. | Percentage of high‑risk provinces with full emergency stockpile (target: 100% in 1 year). |
|  |  |  |  |  | 3. Sign a framework agreement with at least one alternative supplier (domestic or international) for each essential diabetes medicine. | Supply chain restoration time after a climate event (<72 hours). |
| 4 | Adequate insurance coverage and financial protection for people with type 2 diabetes in climate high-risk situations | Health Financing | 88.90% | Iran Health Insurance Organisation, Rural and Nomadic Insurance Fund, Supreme Insurance Council | 1. Design a “climate‑triggered insurance package” covering: free glucose test strips when air quality index (AQI) >150 or temperature >40°C; full coverage of tele‑visits during road closures or internet outages. | Number of diabetes patients using the climate‑triggered package (target: ≥50% of insured patients in high‑risk areas). |
|  |  |  |  |  | 2. Define a mechanism for Qard‑al‑Hasan loans or grants for cooling devices (air conditioner, small refrigerator) for diabetes patients under support organisations (Emdad, Behzisti) in hot regions. | Mean out‑of‑pocket expenditure for diabetes care during a crisis month vs. normal month (target: 40% reduction). |
|  |  |  |  |  | 3. Revise outpatient diabetes service tariffs during emergencies (50% increase for clinics operating under crisis conditions). | Time from proposal to official approval of the package (<6 months). |
| 5 | Monitoring equity in payments for type 2 diabetes care and improving access and continuity of services | Health Financing | 83.30% | Deputy for Statistics & IT (MOHME), Plan and Budget Organisation, Statistical Centre of Iran | 1. Add two questions to the national Household Income and Expenditure Survey: “How much did you spend on diabetes medicines, tests, and visits in the last 3 months?” and “Have you experienced any unmet need for diabetes care due to climate conditions (heat, flood, dust) in the past year?” | Availability of disaggregated provincial data in MOHME information system (binary). |
|  |  |  |  |  | 2. Create an equity dashboard at each Medical University comparing the diabetes cost‑to‑income ratio between high‑ and low‑climate‑vulnerability areas; update quarterly. | Number of universities with active equity dashboard (target: 100% in 2 years). |
|  |  |  |  |  | 3. Hold quarterly feedback meetings with patient associations and diabetes NGOs to adjust financial policies. | Change in Theil/Gini coefficient for diabetes expenditure across vulnerability zones (target: 20% reduction). |
| Key items with highest agreement (100% in Round 1) |  |  |  |  |  |  |
| 6 | Integrated governance and intersectoral collaboration with strong political commitment | Leadership & Governance | 100% | Secretariat of Supreme Council for Health and Food Security, Deputy for Health (MOHME), Department of Environment, Iran Meteorological Organisation, Plan & Budget Organisation | 1. Establish a “National Climate and Diabetes Task Force” with MOHME, Environment, Meteorology, Interior, and Plan & Budget Organisation by end of current year. | Task force established (binary). |
|  |  |  |  |  | 2. Draft a 5‑year intersectoral Memorandum of Understanding defining roles for prevention, preparedness, response, and recovery. | MoU signed (binary). |
|  |  |  |  |  | 3. Secure cabinet approval for an “Operational Plan on Diabetes and Climate Change” with a dedicated budget line. | Operational plan approved and communicated to provinces within 6 months (binary). |
| 7 | Establishing climate-resilient infrastructure to ensure continuity of services during crises | Service Delivery | 100% | Deputy for Management Development (MOHME), Deputy for Treatment (MOHME), Medical Universities | 1. Retrofit diabetes units in 20 selected health facilities in high‑risk provinces with: emergency power (generator or solar inverter) for insulin refrigerators, cooling spaces for patients during heatwaves, flood‑resilient access routes. | Number of retrofitted diabetes centres (target: 100 centres in 3 years). |
|  |  |  |  |  | 2. Produce and distribute “diabetes preparedness kits” in 10 cities with the highest heat index (portable insulin cooler, extra test strips, illustrated guide). | Percentage of diabetes centres with functional emergency power (target: 90%). |
|  |  |  |  |  | 3. Design alternative medicine delivery routes (in cooperation with Red Crescent and Basij) for road‑closure emergencies. | Maximum service interruption time during a climate event (<48 hours). |
| 8 | Training on climate–health interactions and crisis management | Health Workforce | 100% | Human Resources Training Centre (MOHME), Deputy for Education, Family Medicine Department | 1. Design an 8‑hour module “Diabetes and Climate Change: from Diagnosis to Crisis Management” for GPs, nurses, and community health workers; incorporate into annual CME programmes. | Number of trained health workers (target: 50% of staff in high‑risk areas within 2 years). |
|  |  |  |  |  | 2. Conduct a Training‑of‑Trainers (TOT) course for 500 selected staff across three national training hubs (Tehran, Shiraz, Mashhad) within 1 year. | Pre‑/post‑training knowledge test score improvement (≥40%). |
|  |  |  |  |  | 3. Produce multimedia educational content (videos, brochures, simple app) for health workers and patients. | Number of active TOT trainers per province. |
| 9 | Early‑warning and public alert systems | Health Information Systems | 100% | Centre for Communicable & Non‑Communicable Disease Control, IT Centre (MOHME), Iran Meteorological Organisation | 1. Integrate climate alert data (temperature >40°C, AQI >150, dust storm forecast) into the national “190” telephone response system; send SMS alerts to diabetes patients registered in the “Sina” system. | Number of SMS alerts sent and successful delivery rate (target: 80% of registered patients). |
|  |  |  |  |  | 2. Design an automatic algorithm to alert the local health team via their dashboard at least 24 hours before a climate event. | Time from alert trigger to dashboard notification (<2 hours). |
|  |  |  |  |  | 3. Define a “Diabetes Risk Day” index (combining temperature, humidity, and air pollution) and include it in daily meteorological reports. | Diabetes Risk Day index included in daily weather output (binary). |
| 10 | Resilient supply chain for medicines and medical equipment | Medical Products, Vaccines & Technologies | 100% | Iran Food and Drug Administration, Pharmaceutical distribution companies, Emergency warehouses | 1. Perform vulnerability mapping for 10 essential diabetes medicines (insulin pen/vial, metformin, glibenclamide, acarbose, pioglitazone, test strips, insulin syringes) in three pilot provinces (Khuzestan, Hormozgan, Sistan) within 4 months. | Number of essential medicines covered by vulnerability mapping (target: 10). |
|  |  |  |  |  | 2. Deploy online tracking (RFID/barcode) for insulin stocks in regional warehouses; update in “Titak” system daily. | Percentage of regional warehouses with active online tracking (target: 100% in 18 months). |
|  |  |  |  |  | 3. Sign strategic agreements with private distributors and local manufacturers for alternative supply in case primary producer fails. | Alternative supplier agreement in place for each essential medicine (binary). |
